# Automated semi-real-time detection of muscle activity with ultrasound imaging

**DOI:** 10.1007/s11517-021-02407-w

**Published:** 2021-08-16

**Authors:** Anna J. Sosnowska, Aleksandra Vuckovic, Henrik Gollee

**Affiliations:** School of Engineering, University of Glasgow, Glasgow, G12 8QQ UK

**Keywords:** Biofeedback, Electromyography, Muscle contraction, Muscle training, Ultrasound imaging

## Abstract

Ultrasound imaging (USI) biofeedback is a useful therapeutic tool; however, it relies on qualitative assessment by a trained therapist, while existing automatic analysis techniques are computationally demanding. This study aims to present a computationally inexpensive algorithm based on the difference in pixel intensity between USI frames. During an offline experiment, where data was analyzed after the study, participants performed isometric contractions of the gastrocnemius medialis (GM) muscle, as executed (30% of maximum contraction) or attempted (low force contraction up to a point when the participant is aware of exerting force or contracting the muscle) movements, while USI, EMG, and force data were recorded. The algorithm achieved 99% agreement with EMG and force measurements for executed movements and 93% for attempted movements, with USI detecting 1.9% more contractions than the other methods. In the online study, participants performed GM muscle contractions at 10% and 30% of maximum contraction, while the algorithm provided visual feedback proportional to the muscle activity (based on USI recordings during the maximum contraction) in less than 3 s following each contraction. We show that the participants reached the target consistently, learning to perform precise contractions. The algorithm is reliable and computationally very efficient, allowing real-time applications on standard computing hardware. It is a suitable method for automated detection, quantification of muscle contraction, and to provide biofeedback which can be used for training of targeted muscles, making it suitable for rehabilitation.

**Graphical abstract:**

Biofeedback session based on ultrasound imaging (USI) during muscle training. Novel, computationally inexpensive algorithm based on the difference in pixel intensity between USI frames is used to process the video and provide quantitative feedback on the strength of muscle contraction.

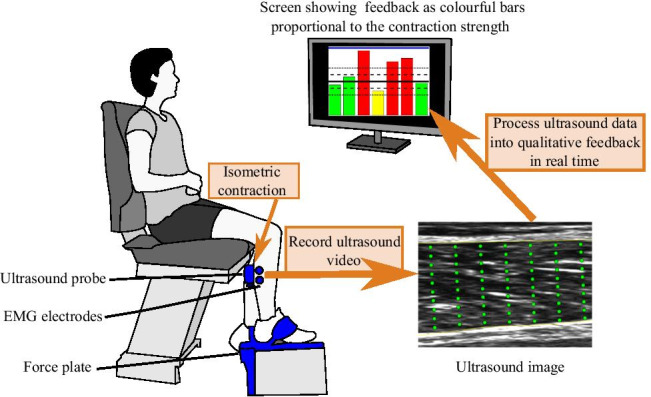

## Introduction

Ultrasound imaging (USI) is a widely used technology in medicine and research. It is a powerful tool, since it is noninvasive, cost effective, and portable, and it has the potential to objectively assess the functionality of muscles and assist in rehabilitation of patients recovering from a range of neuromuscular disorders [1, 2].

In recent years there has been great interest in studying USI to characterize muscle activity since it enables visualization of the muscoskeletal system and evaluation of dimensional properties of the muscles at rest and during contraction [3–7].

Many previous investigations involved manual assessment of US videos, including measurements of fascicle length and pennation angle, in a sequence of US images for analysis of muscle movement [8, 9]. However, manual assessment is time-consuming, subjective, and in general has poor accuracy and repeatability [10, 11]. As a result, work has been undertaken to make the process more automated through the development of mathematical algorithms for feature extraction and tracking. Methods based on feature tracking between ultrasound images with optical flow or cross-correlation [12–14] and feature detection in a single US image [10, 15, 16] have been validated for several applications. These include detection of contracting muscle regions [15] and the study of various muscle architecture changes, such as cross-sectional area, muscle fascicle orientation [16], length [12, 16, 17], intra-fascicular strain, and shearing of aponeuroses [18].

These automated methods are more objective than manual analysis, removing subjective bias; however, processing requires very long computational time, ranging from minutes [14] to hours [13], to analyze video recordings of only 10-s duration. Zhou et al. also reported that the average computation time needed for the identification of parameters in a 300 × 300 pixel frame was about 18 s for the first frame, and 9 s for each subsequent frame that used information obtained previously [19], meaning that analysis would take about an hour for a 10-s recording at 40 fps (frames per second). Farris et al. claimed that when using their UltraTrack software to analyze a 9.2-s long video consisting of 801 frames, it took 17.1 s to load the MAT file with pixel intensity data and 110.3 s to process it with the affine flow algorithm (as run on MacBook Pro with a 2.5-GHz Intel core i5 processor, 8 GB of RAM, and Mac OS X 10.9.5 (Mavericks)), whereas loading an AVI file of 441 frames directly took 96.3 s (Intel core i5 processor, 8 GB RAM) [20]. Because of their relatively high computational complexity, these algorithms can currently only be used for offline applications or require a specialized hardware.

In this paper, we present a fast, computationally inexpensive technique for the precise detection of the onset of contraction and quantification of muscle contractions in terms of the movement of muscle fibers in relation to the maximum contraction. It is based on the comparison of pixel intensities between frames of B-mode ultrasound recordings. We experimentally evaluated this algorithm in experiments involving isometric contractions of the gastrocnemius medialis (GM) muscle. First, the algorithm’s accuracy and repeatability were verified offline by comparing the detections of muscle contractions with recordings of torque and electromyography (EMG). We then analyzed its online performance in an experiment where semi-real-time feedback of the contractions was given.

## Methodology

The investigation described here consisted of two experiments, one where analysis was done offline and the other involving online analysis. During the offline experiment, participants performed isometric contractions of the gastrocnemius medialis (GM) muscle, as executed (30% of maximum contraction) or attempted (low force contraction up to a point when the participant was aware of exerting force or contracting the muscle) movements. During these contractions, USI, EMG, and force data was recorded, which was analyzed after the study (offline). The purpose of this work was to develop an automated algorithm that would be able to detect an onset and quantify muscle contractions. Furthermore, the aim was to verify the sensitivity of USI in relation to EMG and torque data.

The developed method of USI video analysis was then adapted for real-time applications to provide biofeedback based on USI measurements of muscle activity. During the online experiment, participants performed GM muscle contractions at 10% and 30% of maximum contraction, while the algorithm provided visual feedback proportional to the muscle activity during the maximum contraction as recorded with USI.

In this section, the methods for processing and analyzing USI videos are described, followed by the processing methods used for EMG and force data. Details of the offline and online studies, including protocols and participants information, are then presented. The adaptation of the USI processing method for real-time feedback applications is clearly depicted.

The experimental procedures described here were approved by the University of Glasgow Ethical Committee and were performed in accordance with the Declaration of Helsinki. All participants gave written informed consent.

### Ultrasound video processing

The method of ultrasound video processing presented here is based on comparing the intensity of the pixels between video frames.

#### Preprocessing

**Fig. 1:**
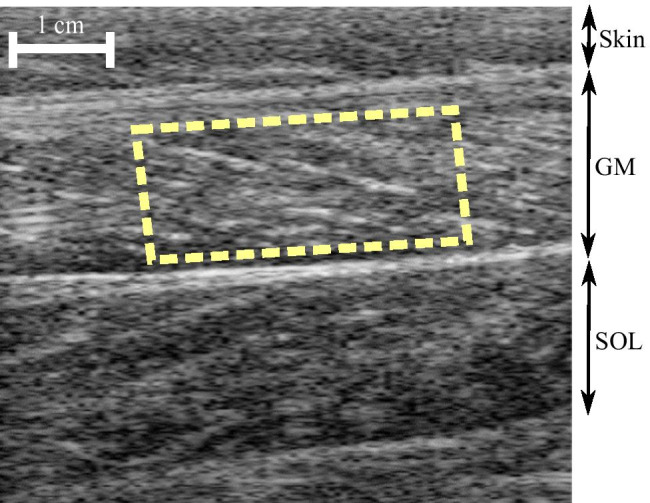
Region of interest selection using a polygon (dashed trapezoid) to include the biggest portion of the muscle without aponeuroses. US image shows scanning region including skin, gastrocnemius medialis (GM), and soleus (SOL) muscles

The individual frames of the ultrasound videos in AVI format were extracted and converted from RGB to grayscale values (255 levels, scaled to the range [0, 1]). The region of interest (ROI) was selected manually using a polygon (trapezoid) containing only the muscle of interest (i.e. the gastrocnemius medialis muscle) and excluding the aponeuroses, as shown in Fig. 1. The ROI selected on the first frame of the initial recording was used for all subsequent recordings for this participant during the session.

#### Ultrasound video analysis

The analysis algorithm can be summarized as follows: Given a matrix $A(i)$ containing all the pixels in the $i$ th USI frame and $M\times N$ representing the dimensions of the ROI, the selected region containing the muscle image was represented by:

1 $${A(i)}_{M\times N}=A(i)\cap {ROI}_{M\times N}$$

Values of each pixel within the ROI were then subtracted between adjacent frames:

2 $$\Delta A{\left(i\right)}_{M\times N}=A{\left(i\right)}_{M\times N}-A{\left(i-1\right)}_{M\times N}$$

For each frame, the absolute value of differences $\Delta A{\left(i\right)}_{M\times N}$ for each pixel was summed over the entire ROI and normalized by the number of pixels, resulting in the normalized pixel difference, $NPD(i)$, of the $i$ th frame,

3 $$\mathrm{NPD}(i)=\frac{{\sum }_{n=1}^{M\times N}\left|\Delta A{\left(i\right)}_{n}\right|}{M\times N}$$

**Fig. 2:**
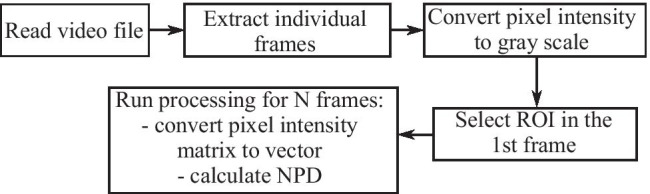
Flow chart of USI video processing method to calculate normalized pixel difference (NPD)

The $NPD$ can be interpreted as a measure of relative change of muscle state between frames. A summary of the USI video processing algorithm is shown in Fig. 2.

Since the intensity of the noise varied with the image intensity, several steps were implemented to deal with the speckle noise in the ultrasound images. The method described here for calculation of normalized pixel difference includes a calculation of the average pixel intensity across the entire image. Therefore, the intrinsic speckle noise of the ultrasound frames would effectively cancel out enabling a robust comparison between the consecutive USI frames.

### Automatic detection of activity

#### Detection of muscle contraction based on USI

**Fig. 3:**
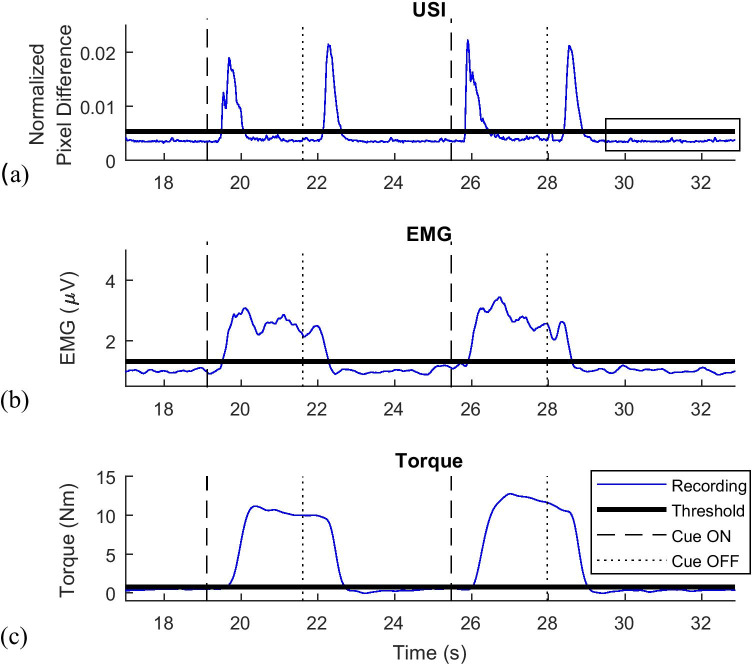
(**a**) USI recordings taken during executed movement trials for a representative participant. The threshold for automated contraction detection is marked. The rectangle marks the resting period between the contractions. (**b**) EMG recordings taken during executed movement trials for a representative participant. The threshold for automated contraction detection is marked. (**c**) Torque recordings taken during executed movement trials for a representative participant. The threshold for automated contraction detection is marked

The threshold of muscle activation was determined based on a recording of the muscle at rest during a baseline period. It was observed that there was always some temporal fluctuation in the signal due to physiological artifacts, such as periodic activity of the capillaries and changes in the muscles even when at rest, which can be seen in Fig. 3(a). In order to account for these, the threshold for muscle activation was determined as the mean + 3 standard deviations (SD) across the baseline period. Since the signal obtained from USI processing had stochastic characteristics and known dynamics, it was possible to apply a similar method for threshold specification to that commonly used in EMG processing [3, 21]. The selection of this threshold value was further supported by the verification that no more than 1.5% of data points during rest exceeded the threshold (average of 1.29 ± 0.23%). This threshold was automatically applied to all recordings.

Plotting the NPD signal over time (Fig. 3(a)), two peaks could be distinguished for each contraction. The first peak corresponded to muscle contraction, whereas the second peak was associated with muscle relaxation. The peaks originated from a large change of pixel intensity within the ROI between subsequent US video frames, indicating muscle movement: The aponeuroses sheared against each other and the pennation angle of muscle fascicles changed, leading to large changes between frames. When the muscle was in a contracted state, there was little activity in the image due to only small movements of the fibers, related to maintaining tension.

In order to automatically detect a muscle contraction, two consecutive peaks exceeding the threshold had to be registered following an action execution cue (Fig. 3(a)). For the first peak, when the contraction was expected, the algorithm found the instances when the signal exceeded the threshold and monitored a subsequent period of 0.25 s. It was the minimum duration of the peak when the muscle was contracted. If within that interval, 70% of the samples stayed above the threshold; then the first detected point was treated as the onset of the contraction. This approach was taken to ensure that the signal exceeding the threshold was not due to random variability of the signal that was seen during baseline recordings. The value of 70% was heuristically determined when analyzing weak contractions as within 0.25 s from the first detection, some samples could still fall below the threshold. Using a higher value would omit detections of actual muscle activity (7.3% of trials) as verified by comparing with USI video frames during a feasibility study.

When looking for the second peak, corresponding to the relaxation, the continuous interval when the signal stayed below the threshold was identified (when muscle was tensed), and then the same condition for peak detection was applied.

The detection algorithm can be summarized as follows:

4 $$\begin{array}{c}if\;NPD\left(i\right)>T\wedge70\%\left(NPD\left(i\right):NPD\left(i+10\right)\right)>T\\i=i_{onset}\\\begin{array}{c}if\;NPD\left(i_2\right)>T\wedge70\%\left(NPD\left(i_2\right):NPD\left(i_2+10\right)\right)>T\\i=i_{offset}\end{array}\end{array}$$

Here, *T* is the threshold value and *i* is the sample number of the USI frame. Onset and offset subscripts stand for the beginning of the contraction and the relaxation phase respectively. The sampling rate of the recordings was 40 fps; thus, 10 samples represent 0.25 s.

#### Detection of EMG and torque activity

The detections of EMG and torque activity followed the same methods as the detection of muscle contraction described above.

For the detection of muscle activations, EMG during a task was compared with the baseline signal recorded at the start of the session. The EMG onset of muscle activation was defined as the time when the enveloped EMG signal exceeded a threshold of mean + 2SD of the EMG signal at baseline (rest). Similarly, the torque onset was detected when the smoothed torque signal exceeded a threshold defined as the mean + 2SD of the torque at baseline [3, 21]. If the signal remained above the threshold for 1 s, the time stamp of the first data point crossing the threshold was considered the moment of muscle contraction. The period of 1 s was selected to ensure that a lasting contraction occurred and that any noise from the signal was not falsely interpreted as muscle activation.

### Offline experimental study

#### Experimental setup and protocol

Eighteen able-bodied participants (age 27.3 ± 6.8 years, 11 male) in self-reported good health with no known sensory or motor deficits took part in the offline experiment.

Participants were facing a 19″ computer screen positioned at eye level approximately 1 m away where the cues to initiate the tasks were displayed. The visual angle of stimuli was 9 degrees. The participant was comfortably seated on a chair, with the dominant leg bent at the knee at approximately 90 degrees and the foot resting on a force plate (FP). The heel was supported and the foot was restrained with Velcro straps to restrict ankle movement. Torque output, EMG, and ultrasound videos were recorded simultaneously.

During the experimental session, participants performed cued motor tasks by pressing on a stationary force platform with their foot (ankle plantar flexion) while contracting the gastrocnemius muscle (isometric contraction), performing either *attempted movements* (AM) or *executed movements* (EM). The AM task was a movement with minimum bodily awareness of performing a physical action during which the participant was instructed to initiate the overt action only up to a point when they became aware of exerting force or contracting the GM muscle. During the EM task, the participant was asked to aim for a contraction force of 30% of their maximum voluntary contraction. This value was chosen to avoid fatigue and because of the relatively high sensitivity of ultrasound at weaker muscle contractions [3].

Initially, baseline measurements were recorded for 120 s when the participant was not performing any movements. Following this, their maximum voluntary contraction (MVC) was measured when the participant pressed on the force platform as strongly as possible 3 times for a period of 2.5 s. Participants rested for 7.5 s between each MVC attempt.

Before each task, participants had a familiarization period lasting for 1 min. During this time, they received visual feedback on their torque output.

Ninety cues lasting 2.5 s each were shown for both the AM and the EM tasks. Immediately after the cue appeared, the participant performed plantarflexion, sustaining the isometric contraction for 2.5 s and relaxing when the cue disappeared. A variable inter-trial (resting) time of 3.0–5.5 s was used to avoid preparation for movement due to habituation with fixed time intervals. The tasks were performed in 120-s long sub-sessions (5 sessions of 18 trials for each task) which allowed the participants to remain alert and avoid fatigue.

#### Offline data acquisition and preprocessing

During the experiments, an ultrasound probe (linear array LV7.5/60/96, central frequency of 6 MHz connected to Echoblaster128, Telemed, Lithuania) was positioned over the belly of the GM muscle. It was aligned to the mediolateral midline of the muscle at the level of the mid-belly to minimize errors due to probe orientation. The probe was placed in a custom-made holder and secured with a Velcro strap around the leg to minimize probe movement relative to the skin. All recordings were performed in B-mode at an average rate of 40 fps with the EchoWave II software (Telemed, Lithuania).

The ankle torque was recorded with a custom-made force platform at 1000 Hz (DAQcard-6024E, National Instruments, USA). The data was acquired in Simulink (MATLAB R2014a, The MathWorks Inc., USA). Prior to analysis, the torque data was smoothed with a moving average filter over 0.01 s, which was symmetric and centered so as not to distort the phase or timing of the signal.

EMG data was recorded at 1200 Hz (g.USBamp, g.Tech, GmbH, Austria) using bipolar Ag/AgCl electrodes positioned over the GM muscle, while the reference electrode was positioned over the ankle. The EMG signal was band-pass filtered between 5 and 500 Hz with a 5th order Butterworth filter within the g.USBamp device, and acquired in Simulink. The raw EMG data was full-wave rectified to produce a linear envelope of the original signal. The data was smoothed with a moving average filter over 0.01 s [22].

A digital output signal from the ultrasound system was used to synchronize data collection between the ultrasound, torque, and EMG measurements.

### Online experimental study

#### Experimental protocol

**Fig. 4:**
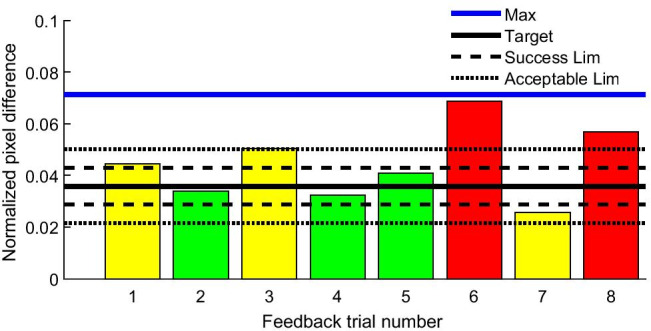
An example of US visual biofeedback during weak (10% MVC) contraction task. The green color presents the “successful” limits of ± 10% of the target value and the yellow color presents the “acceptable” limit of ± 20%, while values outwit “acceptable” range are present in red color

Fifteen able-bodied volunteers (age 30.7 ± 10.8 years, 7 male) participated in the online experiment. Each participant completed two sessions on different days. Prior to each session, the baseline and MVC were recorded, as in the offline experiment. The participant performed weak (10% of MVC) and medium (30% of MVC) contractions of the GM muscle. Each task consisted of 15 cue-based trials (5 trials with real-time dynamometer feedback, shown as a signal progressing in time to teach participants to produce a contraction with a desired intensity, and 10 trials with USI feedback provided after the contraction attempt), lasting 12 s each (2 s rest, 1 s preparation, 4 s execution, 5 s rest). After finishing each trial, the USI-based biofeedback was displayed on the screen in the form of a bar, the height of which was proportional to the value of the detected muscle contraction as shown in Fig. 4.

#### Online data acquisition and preprocessing

The experimental setup was similar to that of the offline experiment, but no EMG data was recorded. The US data was recorded in the same way as in the offline experiment, while a dynamometer (System 3-PRO. Biodex Medical Systems Inc., USA) was used to record torque data.

#### Online ultrasound video processing

The relatively low computational complexity of the proposed algorithm (see Sect. 2.1) enabled the USI videos to be processed in MATLAB in semi-real-time.

For the online processing, the offline processing method was modified to allow access to a raw USI video file (TVD format) that was automatically converted to a binary (BIN) file. A custom C +  + program was used to extract information on the pixel intensity of each frame. Following that, computations similar to the offline algorithm were performed, allowing detection of movement in semi-real-time. Prior to the session, a ROI was selected manually and kept constant throughout the entire session.

**Fig. 5:**
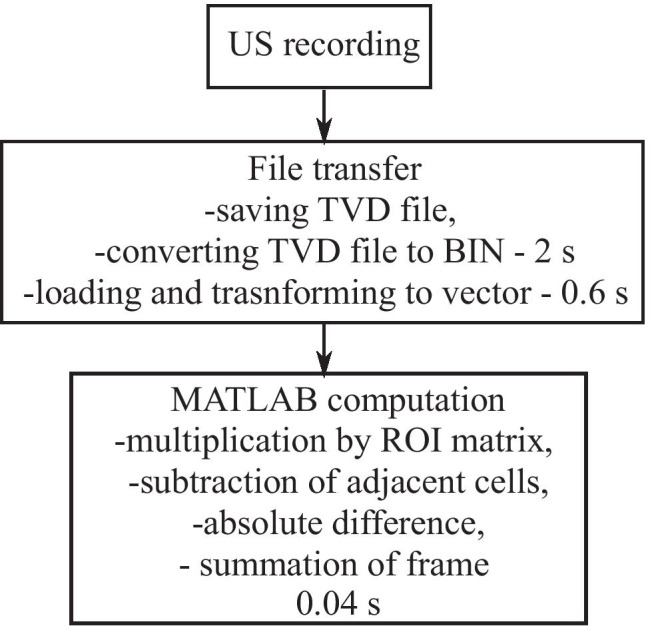
Computational complexity of the processing algorithm and time required for calculations during the online processing. Total processing time is less than 3 s

A breakdown of the approximate times for file transfer and processing of a video with 400 frames (10 s recorded at the rate of 40 fps) is shown in Fig. 5. The values were obtained using MATLAB Profiler.

During the feedback training, 5 s long USI videos (containing 1 s of preparation and 4 s of execution) were recorded. Automated processing of the video occurred during the following relaxation period when the participant was finishing the task and relaxing the muscle. This enabled the feedback to be displayed immediately after the entire trial finished.

#### Ultrasound biofeedback training

For the USI biofeedback training, the threshold of muscle activation was selected as described in Sect. 2.2.1. In order to provide feedback on the contraction intensity, the peak value of the NPD signal during the trial was used, since this allowed distinction between weak and medium contraction intensities and provided information on muscle activity.

The peak NPD value of the muscle contraction recorded during training trials using force platform feedback served as a target for trials when USI feedback was provided. The NPD value was normalized to the result obtained during the MVC test, which indicated the maximum displacement of the muscle fibers during contraction. If the NPD value recorded during the feedback session was within 10% of the target, the trial was considered successful. If it differed by up to 20%, it was deemed acceptable. The outcome was presented to the participants in the form of bars, with colors indicating whether the trial was successful (green), acceptable (yellow), or unsuccessful (red) (Fig. 4).

### Offline and online outcome measures

To determine the robustness of the offline automated USI analysis method, the ability to detect the muscle contractions was compared between USI, EMG, and the force plate (FP). For each participant, the total number of detections made consistently by each measurement method was determined and compared between methods. This approach also enabled comparison of the sensitivity of the different detection methods.

For the online experiment, we analyzed the time required to present the feedback to the participants and the distribution of the trials, i.e., the difference from the target of each attempt across two training sessions. For this calculation, the NPD peak value of each trial was normalized to the NPD peak value recorded during the MVC test prior to the feedback training.

### Statistical analysis

The statistical analysis was performed with the IBM SPSS Statistics v. 24.0.0.0 software using the number of detections made with USI, EMG, and FP for individual participants. Since the data was not normally distributed, as determined by the Shapiro–Wilk test, a non-parametric Friedman test (paired sign rank sum with a significance level of *p* = 0.05) was used to determine whether there were any statistical differences between the detection methods.

## Results

### Offline detection of muscle activity with USI

**Fig. 6:**
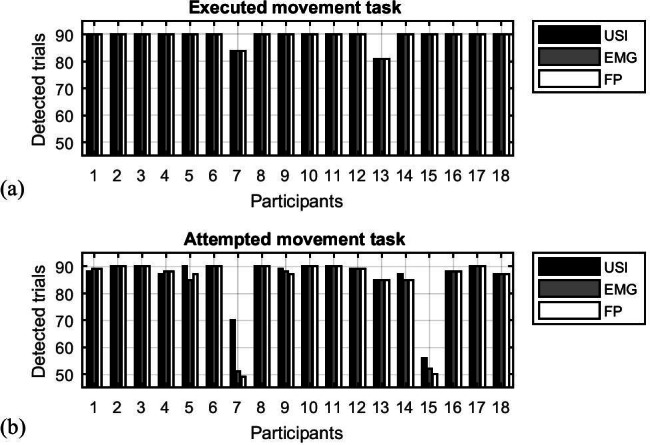
(**a**) Number of detections made with USI, EMG, and force plate (FP) made for each participant during executed movement trials. The total number of trials was 90. (**b**) Number of detections made with USI, EMG, and force plate (FP) made for each participant during attempted movement trials. The total number of trials was 90

During the EM task, the performance of each participant was rated consistently with all three methods, with only two out of 18 participants failing to react to some of the experimental cues (Fig. 6(a)). During the AM task (Fig. 6(b)), bigger discrepancies were observed, with three participants performing real contractions for less than 70% of the cues and only eight reacting with a detectable contraction during all 90 trials. For some participants, all detected contractions were seen with all three methods (USI, EMG, and FP), whereas for others, the contractions were so subtle that EMG and the force plate could not detect these muscle activations.

**Fig. 7:**
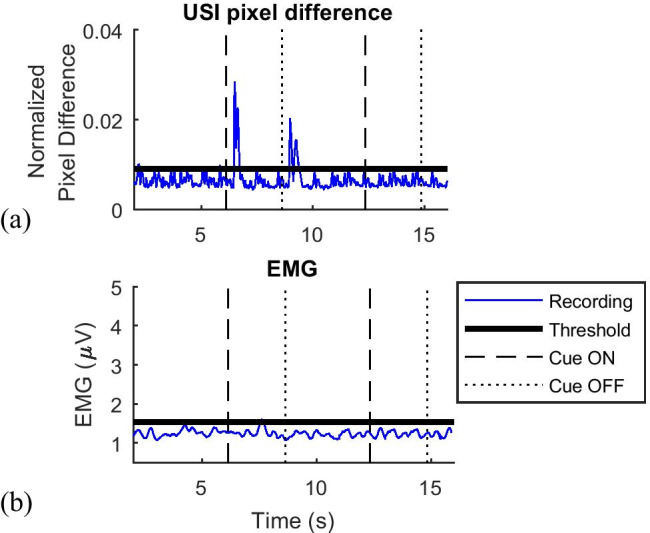
(**a**) Detection of muscle contraction with USI pixel difference and EMG during an attempted movement (AM) task for a representative participant. (**b**) The contraction is only visible with USI. Detection of muscle contraction with EMG during an attempted movement (AM) task for a representative participant. The contraction is only visible with USI

Furthermore, for the instances when the detections differed between the methods, USI detections were reviewed to verify the presence of muscle activity and confirm that real contractions were detected. An example for participant 7 in Fig. 7 show the event during AM when the NPD method registered the contraction (Fig. 7(a)), whereas the EMG signal failed to exceed the threshold (Fig. 7(b)). The similarity in signal morphology between detections made during EM and AM tasks (Figs. 3 and 7) indicated that real muscle activity was detected, which was not seen in EMG recording.

For EM, there was 99.0% agreement between the three methods, whereas for AM, the agreement rate fell to 93.1%. The USI detected 1.35% movements which could not be detected by the other methods, whereas only 0.19% of movements were detected by EMG and force platform but not by USI.

Since data on the detection of muscle contractions and twitches violated the normality assumption, as determined by the Shapiro–Wilk test, a non-parametric Friedman test was used to assess differences in the number of detections made with different methods. Comparing between USI, EMG, and FP, the detections of movements were not significantly different for neither EM nor AM (*p* = 0.662 and *p* = 0.368, respectively).

### Semi-real-time US biofeedback

**Fig. 8:**
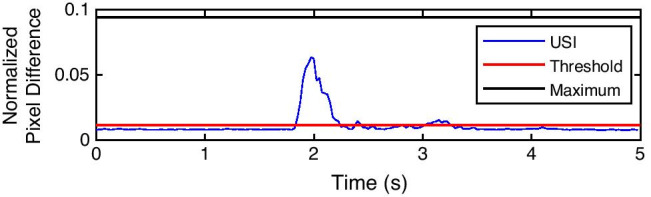
Normalized pixel difference during representative trial of medium contraction

The application of the fast USI processing algorithm made it possible to provide semi-real-time visual feedback on muscle activation proportional to the intensity of the contraction. Figure 8 shows an example of a contraction attempt at 30% MVC. The interval displayed shows the preparation (1 s) and execution (4 s) periods. The peak at around *t* = 2 s indicates the moment when the contraction starts, whereas the relaxation (which would also result in a peak) is not presented in the figure. The height of the peak relative to the MVC indicates the contraction intensity and enables distinction between weak and medium intensities.

**Fig. 9:**
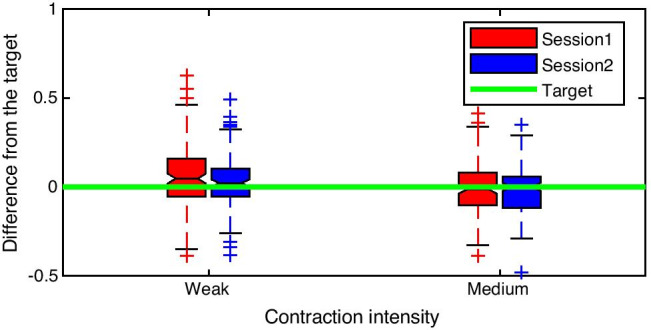
Distribution of attempts for the whole group expressed as difference from the target of normalized pixel difference value relative to peak NPD during the maximum contraction test for medium intensity isometric contraction

The distribution of attempts for both tasks across an entire group is shown in Fig. 9, demonstrating that on average, the participants managed to reach the target consistently, especially for stronger contractions. This indicates the usefulness of USI-based feedback for learning to perform specific movements. Overall, for both sessions, considering the absolute difference from the target relative to the NPD, for weak contractions, the 25% quartile was 0.05, the median was 0.09, and the 75% quartile was 0.17. For medium contraction, these values were 0.04, 0.09, and 0.14, respectively.

Upon completion of each trial, the feedback about the contraction intensity could be shown in less than 3 s (Fig. 7), allowing the participant to adjust the intensity of contraction before the next attempt. On average, the contraction was registered at *t* = 1.13 ± 0.78 s from the beginning of the execution phase (which starts at *t* = 1 s). The execution phase (lasting 4 s) was followed by the relaxation phase (lasting 5 s); therefore, effectively, the feedback was seen about 8 s after the beginning of the contraction, but within 3 s after the trial finished.

To further strengthen the reliability of this method, the following analysis was implemented offline. Two different regions of interest (ROI) were selected (smaller and larger from the original), and the automated analysis was run again to compare the results and classification of trials with originally obtained data. For 13 out of 15 subjects, the results of the feedback sessions remained consistent indicating no influence of ROI, while for the remaining 2 participants, 2 trials or 4 trials (5% and 10% respectively) were classified differently. This further demonstrates the robustness of this method and lack of dependence on the operator or ROI.

## Discussion

In this study, we presented and validated an algorithm for analysis of US videos to detect muscle activity and verified its performance for semi-real-time detections. In the offline experiment, the ultrasound videos recorded from the gastrocnemius muscle during executed and attempted movement were automatically analyzed. These two modalities were selected as they reflect different levels of muscle contraction. They were investigated with a newly developed, computationally inexpensive method based on comparing the intensity of pixels between consecutive frames of the video, to verify its merits for automated contraction detection.

The motivation for using ultrasound imaging to detect the onset and quantify muscle activity originated from its sensitivity and ability to distinguish between the movement of different muscle structures. With the aid of USI, the activity of a specific muscle can be analyzed. Traditionally, EMG or force platform/sensors are used to evaluate muscle activity; however, they do have significant drawbacks that can be overcome by the use of USI. EMG records an electrical signal during muscle contraction showing the electrophysiological features of skeletal muscles and enabling analysis of muscle physiological behavior [23]. Surface EMG (sEMG) provides a relatively simple, noninvasive, and fairly specific way to assess the activation of superficial muscles; hence, it has been widely used in ergonomics, biomechanics, sports science, and kinesiology [24]. Nevertheless, the sEMG signal reflects mostly the activity of the superficial muscles and cannot characterize their morphological properties [25]. It is also susceptible to motion artifacts and crosstalk from distant muscles making it difficult to detect activity from individual muscles [3, 23]. It is possible to record deep muscle activity very precisely with insertion of a needle, but this technique is very local (specific muscle is targeted) and invasive.

Furthermore, the use of a force sensor can also be challenging. Measurements with force plates and dynamometers in general are very useful for determining the torque output that specific muscle can produce during a contraction. However, even with state-of-the-art technology, it is impossible to separate the individual torque output of the relevant muscles and other muscles that can be simultaneously activated, even throughout the rest of the body. An example of this was observed in this study when a participant was able to exert force on the foot plate with the weight of their leg by pushing from the hip, rather than contracting the GM muscle that was being examined. This has also been seen during examination of the force exerted from the shoulder muscles, demonstrating the difficulty in isolating the movement and engaging a single muscle [26].

We showed that by analyzing the normalized pixel difference (NPD) between frames, it was possible to reliably detect muscle contractions. Compared to alternative measurement techniques (EMG and torque), almost no difference (99% agreement) was observed in detection of muscle contractions during the executed movements. Still, the differences seen during the attempted movement task indicate the relevance of using USI for this application. We showed that very subtle muscle activity does not always lead to contractions detected by EMG or the force plate but can be observed with USI. In this study, we did not record statistically significant differences at a group level when detecting the muscle movements with USI, EMG, or force plate, perhaps since the gastrocnemius medialis is also a relatively large, superficial muscle. Still, it should be taken into account that the differences were relevant for individual cases, if not for an entire group. This method could be adapted in the future for use on other muscles which are smaller and deeper, such as the neck or back muscles for which it would be difficult to take measurements with EMG or a force plate. USI has high reliability demonstrated through comparison with EMG data, and high sensitivity allowing very subtle muscle movements to be registered which would be particularly relevant. Thus, it has the potential to be used as an automated method for contraction detection.

It is important to note that through the analysis of the US videos, it is impossible to determine whether the registered muscle movement was active or passive [17]. The movement of the aponeuroses against each other, displacement of the muscle fibers, and shearing against other tissues visible in the image look similar regardless of the type of contraction [27]. In this study, however, no passive movements were performed.

The application of our fast processing algorithm was demonstrated to successfully provide semi-real-time feedback on muscle contraction. The novelty of this lies in the fact that other US analysis methods typically require long computational time not only due to transfer of video files but also due to the computational complexity [13, 14, 19, 20]. The possibility to perform online muscle image segmentation and analysis online has been proposed by Cunningham et al. to study deep cervical muscles [28]. However, such applications require advanced hardware and cannot be performed on standard computers, unlike the computationally efficient algorithm proposed here. It was demonstrated that it can be used on a standard laptop (Intel core i5 processor, 8 GB RAM) and provide the feedback shortly after the contraction attempt. When describing the methods, we separated data transfer time and the processing time showing that with the proposed algorithm, US frames can be processed very efficiently. To minimize overhead, we accessed the raw US data from the device without converting it to AVI format and extracted the pixel intensities directly. In this way, data could then be processed by the algorithm in nearly real time, allowing quantitative feedback on muscle activity to the user. The overall processing time is related to the recording duration and to the frame rate; therefore, modification of the settings or using other ultrasound hardware could affect the processing efficiency. Still, the most significant delay in our algorithm originates from the time taken to transfer the recording from the scanner. Thus, a more efficient hardware setup could lead to significant improvements.

A limitation of our evaluation is that the algorithm has been validated only for one frame rate and for fixed illumination. Adjustments, most likely in threshold specification, might be required if used with other settings. For much higher frame rates, the difference between successive frames might be too small to achieve good signal to noise ratio and might require downsampling. Thus, in the future, the analysis of the baseline period should be performed on videos recorded with a different contrast or using different US scanners. Another limitation of the study was that the activity of other muscles involved in the movement, such as the soleus muscle, was not measured. There is a possibility that the soleus muscle could potentially be activated and initiate the movement of the GM; however, it is quite unlikely to be achieved voluntarily. In addition, due to the relatively large size of the gastrocnemius muscle, crosstalk from surrounding muscles should not be significant.

One application of feedback training of the muscoskeletal system is in neuromuscular rehabilitation, to allow the central nervous system to re-establish appropriate sensory-motor loops under volitional control and regain motor control following injury, disease, or surgery [29, 30]. In this study, feedback was provided at the end of a 5-s trial which consisted of both contraction and relaxation. Feedback time could be considerably reduced to about 2 s if it would detect the maximum of contraction during the recording (e.g., detecting the declining slope in Fig. 8), rather than waiting until the end of a whole trial. Even though the feedback was delayed, it was demonstrated that participants were able to use it to obtain qualitative and quantitative information on muscle contraction intensity. The setup is consistent with MRI and h-reflex studies during which feedback with a temporal delay, i.e., after the trial, was provided leading to improvement in participant performance [31, 32]. If the method described here is to be used in rehabilitation, it is important to highlight that the current algorithm cannot distinguish between the muscle contraction and muscle relaxation. In both cases, the NPD peak has similar characteristics and could therefore introduce some false positives in the studies. In order to account for that, the rehabilitation protocols need to be very precise and clearly indicate when contraction and relaxation should be attempted. Knowing when specific movement is expected and considering the fact that, if starting from rest, contraction would always precede the relaxation, these two actions can be successfully classified.

Image processing methods and automated analysis of USI recordings are affected by the imperfections associated with USI such as speckle noise, signal attenuation or dropout, and trans-planar motion of important parts of the image [14, 28, 29], making the automated processing challenging. In the current study, these problems were addressed through selection of an appropriate threshold of muscle activity.

USI-based feedback is commonly used in rehabilitation and muscle training, with US videos being shown to the participants in real time. Currently, the main applications include learning to activate and control deeper abdominal muscles [33, 34], trunk muscles [35, 36], and pelvic floor muscles [37], with the objective to overcome lower back pain and to stabilize the lumbar region. The exercises lead to improvements in controlling the muscles; however, cues and commentary from a highly trained clinician with extensive USI experience are always necessary to identify the important features. For all these applications, using our automated quantitative biofeedback method of detecting muscle activity would be advantageous.

Currently, USI biofeedback is implemented by trained personnel who base their judgement on visual observation of the video and experience. To use the method proposed here, an operator needs to only be able to position the ultrasound probe over the muscle of interest and identify the relevant region in the first USI frame. No further experience with use of ultrasound or human anatomy is required. Advancement and miniaturization of sensor technology might in the future lead to development of low-cost USI technology that could be used for rehabilitation applications outside the hospital setting. Such applications would require objective quantitative analysis in the absence of a human expert.

In this study, feedback was based only on the maximum value of the normalized pixel difference during a contraction. Depending on the application, other USI-derived features, such as the area under the NPD signal during an entire contraction, might give a better indication of the total contraction intensity.

It would also be possible to apply the algorithm to study and train other deeper muscles, for example, in the neck or shoulder. In this case, since it would not be possible to provide initial training with torque feedback, an initial input from a trained person would be necessary. Alternatively, the method could be used, for example, solely to detect small muscle activity, which would be advantageous in rehabilitating the patients recovering control over the muscles.

## Conclusions

The proposed USI algorithm based on differences in pixel intensity between successive frames was shown to be computationally efficient and provides a detection rate of muscle contraction comparable to electromyography or force measurements. It can be used to provide semi-real-time feedback to train people to achieve a desired level of contraction. Future applications are in rehabilitation of deep muscles where it can reduce therapist time and provide quantitative feedback to improve patient performance.
